# In-Plane and Out-of-Plane MEMS Piezoresistive Cantilever Sensors for Nanoparticle Mass Detection

**DOI:** 10.3390/s20030618

**Published:** 2020-01-22

**Authors:** Andi Setiono, Maik Bertke, Wilson Ombati Nyang’au, Jiushuai Xu, Michael Fahrbach, Ina Kirsch, Erik Uhde, Alexander Deutschinger, Ernest J. Fantner, Christian H. Schwalb, Hutomo Suryo Wasisto, Erwin Peiner

**Affiliations:** 1 Institute of Semiconductor Technology (IHT) and Laboratory of Emerging Nanometrology (LENA), Technische Universität Braunschweig, 38106 Braunschweig, Germany; m.bertke@tu-braunschweig.de (M.B.); wilombat@tu-braunschweig.de (W.O.N.); jiushuai.xu@tu-braunschweig.de (J.X.); m.fahrbach@tu-braunschweig.de (M.F.); h.wasisto@tu-braunschweig.de (H.S.W.); e.peiner@tu-braunschweig.de (E.P.); 2 Research Center for Physics, Indonesian Institute of Sciences (LIPI), Tangerang Selatan 15314, Indonesia; 3 Department of Metrology, Kenya Bureau of Standards (KEBS), Nairobi 00200, Kenya; 4 Fraunhofer Wilhelm-Klauditz-Institut (WKI), 38108 Braunschweig, Germany; ina.kirsch@wki.fraunhofer.de (I.K.); erik.uhde@wki.fraunhofer.de (E.U.); 5 SCL-Sensor.Tech. Fabrication GmbH, 1220 Vienna, Austria; alexander.deutschinger@sclsensortech.com (A.D.); ernest.fantner@sclsensortech.com (E.J.F.); 6 GETec Microscopy GmbH, 1220 Vienna, Austria; chris.schwalb@getec-afm.com

**Keywords:** MEMS piezoresistive cantilever sensors, dynamic mode, carbon nanoparticle, particle mass measurement

## Abstract

In this study, we investigate the performance of two piezoresistive micro-electro-mechanical system (MEMS)-based silicon cantilever sensors for measuring target analytes (i.e., ultrafine particulate matters). We use two different types of cantilevers with geometric dimensions of 1000 × 170 × 19.5 µm^3^ and 300 × 100 × 4 µm^3^, which refer to the 1st and 2nd types of cantilevers, respectively. For the first case, the cantilever is configured to detect the fundamental in-plane bending mode and is actuated using a resistive heater. Similarly, the second type of cantilever sensor is actuated using a meandering resistive heater (bimorph) and is designed for out-of-plane operation. We have successfully employed these two cantilevers to measure and monitor the changes of mass concentration of carbon nanoparticles in air, provided by atomizing suspensions of these nanoparticles into a sealed chamber, ranging from 0 to several tens of µg/m^3^ and oversize distributions from ~10 nm to ~350 nm. Here, we deploy both types of cantilever sensors and operate them simultaneously with a standard laboratory system (Fast Mobility Particle Sizer, FMPS, TSI 3091) as a reference.

## 1. Introduction

Pollutants in the air are often invisible and come from many different sources. Sulfur dioxide (SO_2_), for instance, is emitted from coal-burning activity in power plants and greatly contributes to the increase of particulate matter (PM), i.e., PM_2.5_ and PM_10_. In addition, the incomplete combustions of fossil fuels, biofuels, biomass, and black carbon (BC) [1,2] cause the appearance of fine (FPs) or ultrafine particles (UFPs). Since these particles are suspended in the air as aerosols, they may cause serious health problems. For instance, the UFP, whose diameter is less than 100 nm, can be easily deposited in a respiratory tract and consequently enter deep into the lungs, and thereby adversely affect the central nervous and cardiovascular systems [3,4,5]. It leads to health problems, e.g., lung diseases, heart diseases, decreased lung function, aggravated asthma, and irregular heartbeat [6]. Increased global trends on airborne diseases have necessitated the establishment of air quality standards to mitigate the effects. Therefore, the development of robust devices for continuous monitoring and sensing of airborne particulates is required for ensuring environmental control, quality, and health.

Various particulate sensing systems have been developed in recent years. One such method involves counting particles by measuring the current of a charged particle (connected via a sensitive current meter) [7,8,9]. Furthermore, dynamic light scattering has also been established as a technique for measuring the average particle size and size distribution in a suspension [10,11]. However, on the one hand, the particle charge-based measurement usually needs many electrodes for sensing extended particle-size distributions, and the corresponding instruments are large in size. On the other hand, particle counting based on light scattering method leads to a lower limit of detectable particle size (~0.3 µm) [12]. Another approach is based on gravimetric measurements, in which resonant mass sensors are normally employed as the key monitoring elements that primarily rely on the principle of resonance-frequency shift, e.g., quartz crystal microbalance (QCM) [13,14] and cantilevers [15,16,17]. Gravimetric-based cantilever sensors have high potential and capability to detect small amounts of mass and are widely used in gas sensing [18], fluid density [19], and fluid viscosity measurements [20], etc. Moreover, they have also been employed in contact resonance mode to measure the thickness and rheological properties of thin films [21,22].

In this study, we operate in-house developed cantilever and commercial-based atomic force microscopy (AFM) micro-cantilever sensors for real-time monitoring of carbon nanoparticle mass concentrations. To excite these cantilever beams, both sensors employed an electrothermal actuation mechanism. In this case, power dissipation in a diffused resistor or in a metal strip causes a temperature increase, thereby resulting in the material expansion, which ultimately initiates mechanical actuation [23]. To actuate the in-house fabricated cantilever beam in an in-plane direction, a heating component was placed at the clamped end of the cantilever near the sensing component. It should be noted that the small distance between the heating resistor and sensing component; however, introduces a thermal parasitic coupling (i.e., from the actuating part to the sensing part). To demodulate “the contaminated” signal output and eliminate the parasitic effect, we, therefore, added an optimization process. On the other hand, the AFM micro-cantilever was actuated in an out-of-plane direction. This was realized by placing an imprinted conductor on the free end of the cantilever beam, while the sensing component was positioned at the fixed end. In this condition, there is a sufficiently large distance between the actuating and sensing parts, which results in an optimum resonance response with a negligible direct thermal-parasitic effect. In our study, we have also evaluated the performance of these two sensors in the framework of their quality factor and resolution for nanoparticle mass-concentration sensing. In addition, a standard laboratory system (Fast Mobility Particle Sizer, FMPS, TSI 3091) was used as a reference to measure the concentration of carbon nanoparticles in a sealed chamber.

## 2. Materials and Methods

### 2.1. ElectroThermal Piezoresistive Cantilever Sensor (EtPCS) 

**Figure 1:**
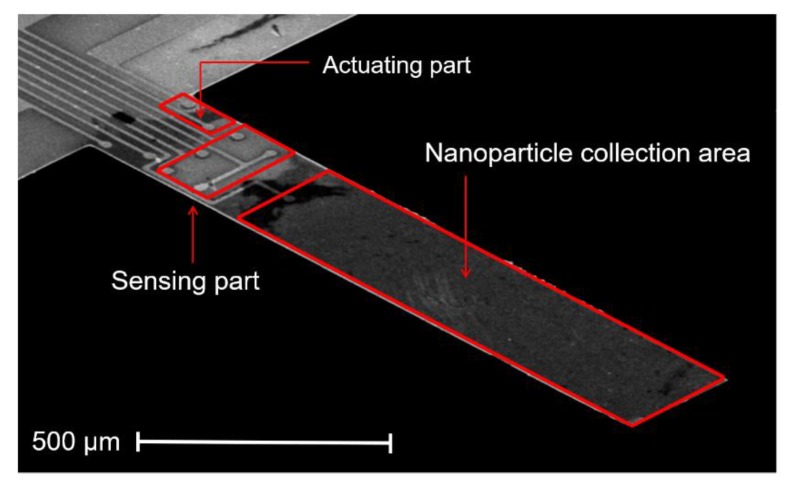
Scanning electron microscopy (SEM) image of the electrothermal piezoresistive cantilever sensor (EtPCS) showing an actuating part and four piezoresistors configured in a full Wheatstone bridge at the clamped end. A *p*-doped area (740 × 170 µm^2^) was created on the free-end of the cantilever beam for the collection of carbon nanoparticles.

An electrothermal piezoresistive cantilever sensor (EtPCS) is designed for in-plane resonant-mode detection. This EtPCS sensor consists of a heating resistor (HR) and four piezoresistors configured in a Wheatstone bridge (WB), which are intended for mechanical actuation and electrical sensing, respectively [12,19,20]. The HR has a resistance of ~992 Ω, while the four resistors of the WB have resistances of ~803 Ω, ~794 Ω, ~860 Ω, and ~847 Ω. The lateral size of each of these piezoresistors is approximately 60 × 10 µm^2^. As shown in Figure 1, the HR is located at the clamped end of the cantilever for efficient actuation in an in-plane bending mode. On the other hand, the WB is also closely located to the HR to facilitate the high-amplitude response of the cantilever due to the high in-plane strain sensitivity of the WB [24]. The length and width of the rectangular cantilever are 1000 µm and 170 µm, respectively, and its thickness is ~19.5 µm, which translates to a mass *m*_0_ of the cantilever of ~7.7 µg. The cantilever’s length-to-width ratio is about 6 and has been examined [25] to yield a high quality factor (*Q* factor) for dynamic operation. These sensors were designed and exclusively fabricated (in the Institute of Semiconductor Technology (IHT)-TU Braunschweig, Germany) using *n*-type silicon-based material by employing bulk micromachining technologies as described in [26,27]. The electrothermal cantilevers offer higher integration levels compared to those counterparts using external piezoelectric actuators for airborne carbon nanoparticle detections, as the sensors can be self-excited and self-readout [28]. The main sensor fabrication processes included thermal oxidation, photolithography, dopant diffusion (phosphorus and boron), etching processes (i.e., HF dip and cryogenic etching of silicon, using inductively coupled plasma-reactive ion etching (ICP-RIE) with SF_6_/O_2_ as process gas mixture), contact-holes formation, and metallization of electrical contacts (Cr/Au = 30/300 nm) followed by a lift-off process. 

**Figure 2:**
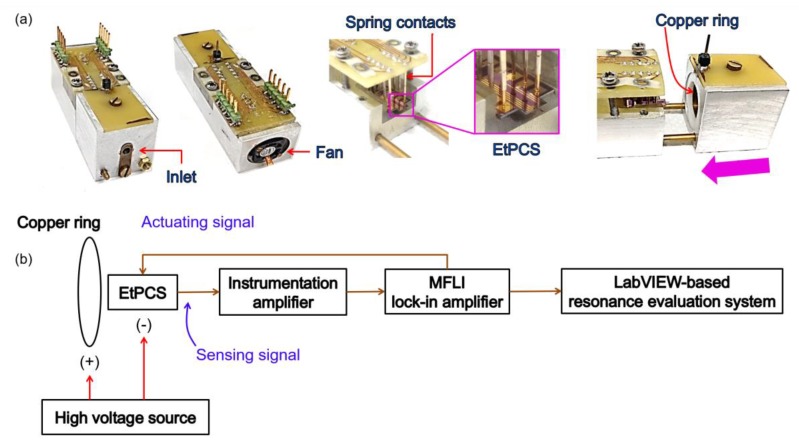
(**a**) Electrothermal piezoresistive cantilever sensor (EtPCS) setup in its housing employing spring contacts for electrical connection. (**b**) Setup to measure the signal output of an EtPCS using an MFLI instrument interfaced with a home-made LabVIEW-based software for tracking its resonance frequency in real-time. For nanoparticle trapping by electrophoresis/dielectrophoresis, a high voltage is applied between a sampling area on the cantilever and its surrounding copper ring.

To evaluate the resonance frequency of the EtPCS, we utilized a lock-in amplifier (type MFLI from Zurich Instrument) as the main hardware system, as illustrated in Figure 2b. The electrical signal generated by the WB is amplified using an external instrumentation amplifier (Analog Devices, AD8421) and is fed afterward into the MFLI. The MFLI instrument provides an application programming interface (API) that allows users to communicate with the hardware through their own software. In this case, we employ our own LabVIEW-based software for evaluating the resonance state of the EtPCS. For nanoparticle collection field, a *p*-type area (~170 × 740 µm^2^) was created on the free-remaining region of the cantilever beam with its boundary close to the WB (as depicted in Figure 1). It was subsequently connected to a negative high direct current voltage, i.e., −115 V. Furthermore, a copper ring (Figure 2a) was connected to +115 V and placed around the cantilever. Consequently, positively charged (and uncharged) nanoparticles would be attracted (and polarized/attracted) to the selected cantilever surfaces by electrophoresis and dielectrophoretic mechanisms, respectively [27,29,30]. This nanoparticle sampling method has been proven to be effective not only for larger silicon microcantilevers but also for smaller nanoscale silicon pillars [17,31,32].

**Figure 3:**
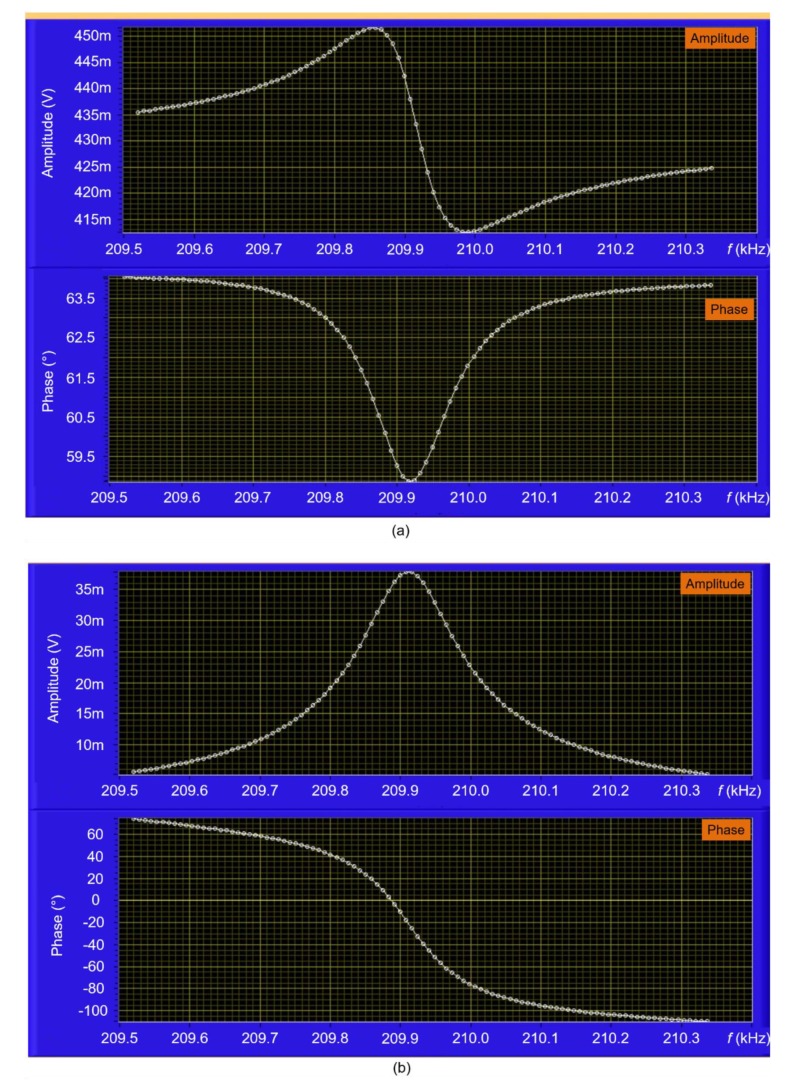
A LabVIEW-based user interface to execute a frequency sweep around the observed resonance mode of the EtPCS. Reference parameters of 0.43 V amplitude and 63.9° phase with the Fano resonance (**a**) are calculated to yield an optimized frequency response (**b**), i.e., a symmetrical amplitude shape and a monotonic phase transition.

**Figure 4:**
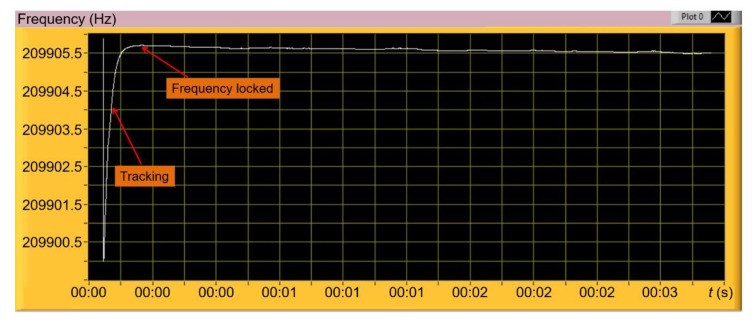
A developed LabVIEW-based user interface to track and lock the resonance frequency of the EtPCS. The selected reference parameters are included for keeping the optimized phase-locking state during resonance tracking.

Due to the existence of direct-thermal parasitic coupling effects on the sensing part resulting in an asymmetric amplitude shape and a reverse phase response (Fano resonance) [30], a reference-signal subtraction method is embedded in our LabVIEW system. This method has successfully been demonstrated to remove the parasitic effect on the EtPCS output signal and construct an optimized frequency response [24,33,34]. In this work, we created a LabVIEW-based system to accomplish a numeral reference parameter for a differential calculation to give an optimized frequency response. In Figure 3a, it is demonstrated that unoptimized frequency response is observed when the reference parameters were set to zero. By addressing the reference amplitude to 0.43 V and using 63.9° for the phase, we could realize an optimized frequency response, i.e., a symmetrical amplitude shape and monotonical phase response (Figure 3b). The reference amplitude was estimated at the mid-point of the maximum and minimum values of the asymmetric amplitude, while the reference phase was determined at a point very close to the phase baseline of the phase response. This numeral reference was then implemented in a phase-locked loop (PLL) system (LabVIEW-based) to keep the optimized state for phase locking and easily facilitate resonance-frequency tracking in real-time (Figure 4).

### 2.2. Atomic Force Microscopy Micro-Cantilever Sensor (AFM-MCS)

**Figure 5:**
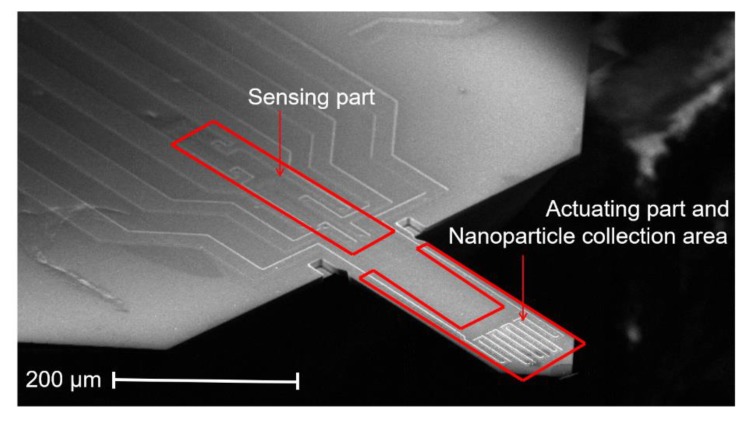
SEM image of the atomic force microscopy micro-cantilever sensor (AFM-MCS) showing the meander-shape thermal bimorph actuator at the cantilever free end and four resistors configured in a half Wheatstone bridge, i.e., two active (i.e., longitudinally strained) resistors and two passive (i.e., unstrained) resistors placed at the clamped end of the cantilever and embedded in the bulk chip region, respectively.

A tip-less atomic force microscopy micro-cantilever (AFM-MCS, PRSA-L300-F80-TL-PCB) beam, fabricated by SCL-Sensor. Tech. Fabrication GmbH comes with an Al meander resistor and an integrated piezoresistive Wheatstone bridge (Figure 5) [35]. The Al meander resistor, whose resistance amounts to ~37 Ω, is embedded between insulating layers and works as a thermal bimorph actuator to trigger an out-of-plane bending mode. Moreover, four integrated *p*-type resistors in the sensing part (with a resistance of ~999 Ω, ~967 Ω, ~1025 Ω, and ~971 Ω) are configured in a half Wheatstone bridge of two longitudinally strained piezoresistors on the cantilever close to its clamping and two unstrained resistors on the bulk area. The length and width of this cantilever are 300 ± 5 µm and 110 ± 3 µm, respectively. Its thickness is ~4 µm, and the mass *m*_0_ of the cantilever amounts, thus to ~0.31 µg, i.e., a factor of ~20 less than the EtPCS.

**Figure 6:**
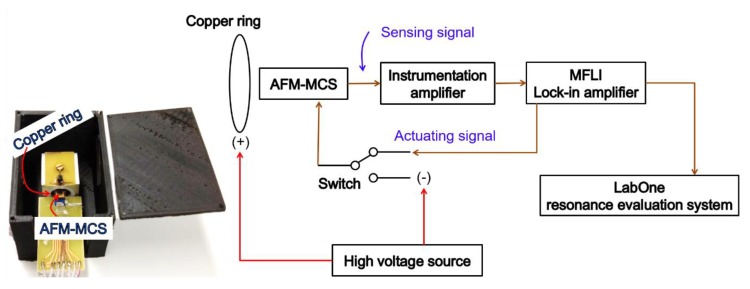
Setup to measure the AFM-MCS signal output using an MFLI instrument (Zurich Instruments AG, Switzerland) interfaced with its default software (LabOne) for observing the cantilever’s resonance frequency by frequency sweeping. A high voltage is applied to the cantilever for nanoparticle trapping by electro-/dielectrophoresis. A manual switch is used to cycle between connecting the cantilever with the actuating signal from a lock-in amplifier and with a high-voltage source for particle collection. During particle measurements, the protection-box cover was opened, as shown in the photograph.

By using the MFLI instrument, we measured the resonance frequency using the default LabOne software of the MFLI, as illustrated in Figure 6. Compared with the EtPCS setup, where the parts for actuating the cantilever into resonance and for particle collection are located in separate areas of the cantilever (cf. Figure 1), the actuating part of the atomic force microscopy micro-cantilever sensor (AFM-MCS) also serves as nanoparticle collection area. Consequently, cantilever-actuation and particle–collection steps were executed successively by using a manual switch. The time duration of both steps, i.e., measurement and particle collection were 80 s and ~1 min, respectively. During the measuring step, resonance frequency was captured in one frequency sweep and not tracked in real-time using a phase-locked loop (PLL). During particle collection, the meander resistor was connected to a negative high voltage of direct current (DC), i.e., −115 V, while a copper ring placed around the cantilever was connected to +115 V of DC.

**Figure 7:**
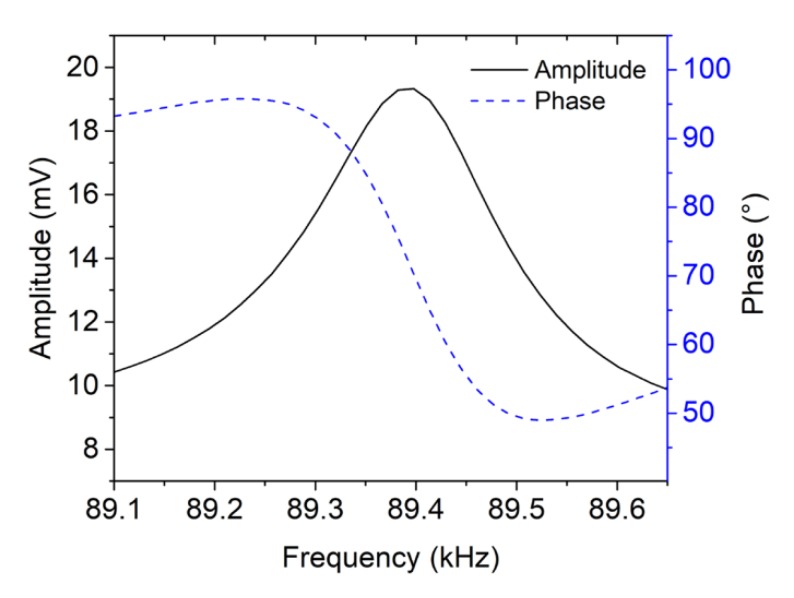
Frequency response of the AFM-MCS before particle exposure exhibiting a resonance frequency at ~89.38 kHz with phase and amplitude of ~72° and ~19 mV, respectively.

We excited the actuating part of the AFM-MCS with AC and DC voltage amplitudes of 200 mV and 1 V, respectively (corresponding to an input power of *P* = 27.3 mW). Figure 7 depicts the amplitude and phase responses of the AFM-MCS upon applying a frequency sweep in the range of 89.1 kHz–89.6 kHz. It shows a maximum amplitude considered as the resonance state at ~89.38 kHz. The corresponding resonance phase is ~72°. Unlike the EtPCS, the frequency response of the AFM-MCS cantilever (Figure 7) shows a symmetrical amplitude shape and monotonical phase response without any additional optimization. For this type of thermal bimorph actuation, the sensing part with piezoresistors is located away from the actuating part, i.e., the meander-shape resistor. Hence, the AFM-MCS shows much less direct-thermal parasitic crosstalk effects. Nevertheless, AFM-MCS cantilever has a smaller amplitude (~19 mV) and a lower *Q* factor of 189 (which is determined by gradient phase fitting [36]) compared to the EtPCS, whose amplitude and quality factor (*Q*) amount to ~0.43 V and ~2000, respectively. 

### 2.3. Nanoparticles Generation

**Figure 8:**
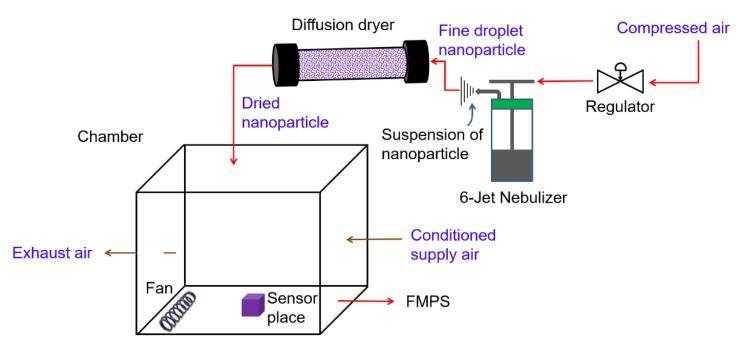
Schematic of the nanoparticle-generation setup as a test chamber, involving a nebulizer, a diffusion dryer, a fan, and a fast mobility particle sizer (FMPS).

An assessment of particle exposure using carbon-based nanoparticles was performed in a 1 m^3^ test chamber made of glass. The test chamber was operated under dynamic conditions, i.e., at a continuous air-flow rate of 10 L/min, a temperature of ~23 °C and relative humidity of ~40–50%. The generation of nanoparticles was started by nebulizing carbon nanopowder (<50 nm, Sigma-Aldrich, Taufkirchen, Germany). Initially, this powder was dispersed in a solution of water and isobutanol using an ultrasonic bath. As illustrated in Figure 8, compressed air (controlled by a regulator) is used to alter the input air pressure that enters a 6-Jet Nebulizer (BGI Inc., Butler, NJ, USA). Inside the nebulizer, the incoming compressed air emits bubbles in the nanoparticle solution and further results in a fine-droplet jet. Furthermore, the droplets (sprayed from the nebulizer) were transferred directly through a diffusion dryer (TSI Inc., Model 3062, Shoreview, MN, USA) to be dried. The diffusion dryer contained a trap for collecting large drops of water and removed excess moisture using desiccants (e.g., silica gel) by diffusion. At the outlet of the dryer, dried nanoparticles were obtained, which were then flown into the sealed glass chamber. Moreover, a ventilation fan was used to circulate the generated nanoparticles inside the chamber [27,28].

## 3. Result and Discussion

To evaluate the performance of the cantilever sensors for nanoparticle mass-concentration monitoring, carbon nanoparticle sampling and detection were conducted under an environment, as shown in the previous section. The nanoparticles generated in a sealed chamber were continuously measured by a standard laboratory particles-measurement system (i.e., Fast Mobility Particle Sizer (FMPS), TSI 3091) to determine the total particle number concentration and its size distribution within 5.6 nm to 560 nm divided into 32 size bins. In addition, we determined mass concentrations of the airborne particles from the number concentrations assuming a constant particle density of *ρ* = 2.26 g/cm^3^ and spherical particle shape according to *ρ* × π/6 × diameter^3^.

A resonant cantilever, as a gravimetric-based sensor, represents a mass-concentration detector. As shown in Equation (1), a calibration factor (*CF*) in units of µg × min/(m^3^ × Hz) is needed to convert the measured resonance-frequency shift rate (Δ*f*_R_/*t*_CT_) into a mass-concentration regime, which is done against the FMPS as a standard reference instrument [27,30]:

(1) $$C_{m}=CF\times \frac{\Delta f_{R}}{t_{\mathrm{CT}}}$$

where *C*_m_ and *t*_CT_ are nanoparticle-mass concentration measured by the cantilever sensor and particle- collecting time (sampling time), respectively. A limit of detection (LOD) of the sensor can be formulated using this *CF* as follows:

(2) $$LOD=\frac{3\times \sigma \times CF}{t_{\mathrm{CT}}}$$

where *σ* is the frequency-noise floor determined over *t*_CT_. 

### 3.1. EtPCS Cantilever Performance

**Figure 9:**
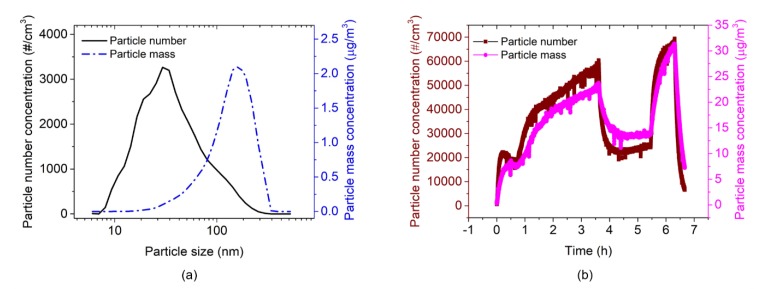
(**a**) Typical particle number and mass concentrations over particle diameter and (**b**) total particle number/mass concentrations over time measured by FMPS.

The EtPCS was operated under a fixed air-flow rate. A constant flow of particle-laden air of 680 cm^3^/min [29] generated by a battery-powered small DC fan was conducted through the small inlet of the sampler. Typical particle size distributions of number and mass concentrations were measured by the FMPS, as shown in Figure 9a, revealing average particle sizes of ~30 nm and ~165 nm, respectively. Corresponding concentrations of particles were measured for ~6.7 h, as delineated in Figure 9b, showing particle number concentrations in the range of approximately 20,000–66,000 #/cm^3^. These particle number concentrations are correlated with mass concentrations in the range of about 7–31 µg/m^3^, which were calculated by:

(3) $$C_{m\_\mathrm{FMPS}}\left(\mathrm{in}\;\frac{µg}{m^{3}}\right)=\frac{\pi }{6}\rho \left(\mathrm{in}\;\frac{\mathrm{kg}}{m^{3}}\right)\times 10^{-12}\times \overset{32}{\underset{i=1}{\sum }}d_{i}^{3}\left({\mathrm{in}\;\mathrm{nm}}^{3}\right)\times C_{n\_\mathrm{FMPS},i}\left(\mathrm{in}\;\frac{\#}{{\mathrm{cm}}^{3}}\right)$$

where *C*_m_FMPS_ and *ρ* denote the total particle mass concentration (measured by FMPS) and particle density, respectively, while *d*_i_ and *C*_n_FMPS,i_ are the particle diameter and particle number concentration in the *i*-th size bin, respectively.

**Figure 10:**
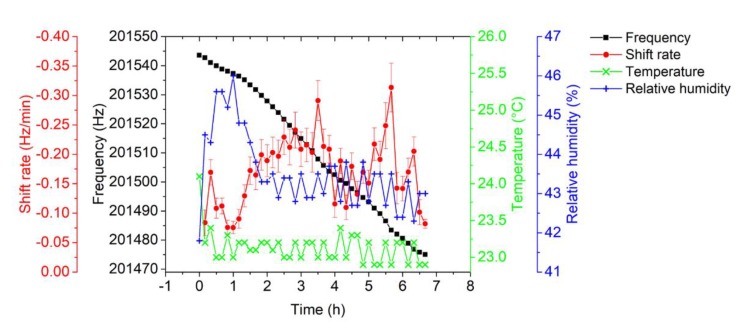
Resonance frequency of the EtPCS tracked by the LabVIEW-based PLL in real-time and the corresponding frequency-shift rate at 10-min sampling of carbon nanoparticles under stabilized ambient conditions (*T* = 23.2 ± 0.19 °C; *rH* = 43.5 ± 0.59%).

Simultaneously, the EtPCS was operated with its resonance frequency locked at −25° and tracked using the LabVIEW-based PLL in real-time at a rate of about 837.1 Sa/s. The resonance frequency decrement due to particle collection and its frequency-shift rate determined over a 10-min sampling time is delineated in Figure 10. In contrary to our previous design [27,30], we did not operate the EtPCS under cyclic switching between separated particle-sampling and frequency-tracking modes but measured frequency shift under continuously maintained particle collection, i.e., at applied high voltage. In addition, fluctuation of temperature and humidity in the chamber were maintained at nearly constant values fluctuating by less than ~0.2 °C and ~0.6%, respectively.

**Figure 11:**
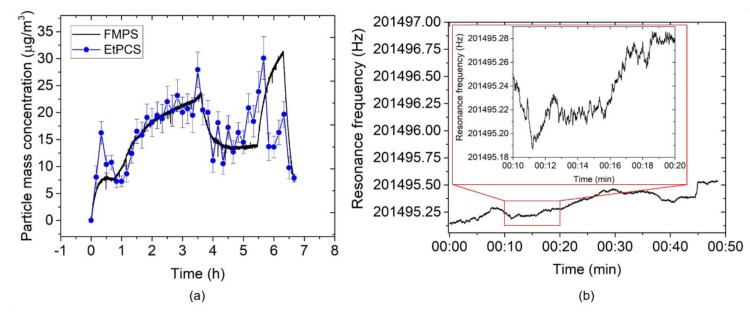
(**a**) Carbon nanoparticle mass concentration measured by the EtPCS (full blue circles) in comparison with FMPS (full black line). (**b**) The frequency stability measurement under a temperature of 22.3 ± 0.4 °C and relative humidity of 23 ± 1%. The inset shows an exemplary detail enlargement of the frequency tracking within 10 min showing *σ* ≈ 27 mHz.

Figure 11a depicts nanoparticle mass concentrations as measured by the EtPCS cantilever in comparison with the FMPS reference. For calculating the mass concentrations from the frequency- shift rate, Equation (1) was used with *CF* = 96 µg×min/(m^3^ × Hz). Within the sampling time of 10 min, an average deviation of 24.9 ± 5.3% relative to the ‘true values’ measured by FMPS was observed at an average uncertainty of 2.3 ± 0.1 µg/m^3^. A much smaller deviation (7.2 ± 1.9%) was achieved in a period of 1 h–3.3 h. Large error contributions were found for the measured values within the first 40 min and at the last 2.7 h. This may have been caused by fluctuations of ambient conditions (*T*, *rH*, particle size distribution) around the cantilever [37,38]. In the previous work [24], we found a resonance frequency decrease of a bare EtPCS of up to ~11 Hz upon an increase of both temperature (~2 K) and *rH* (~14%). From Figure 10, we can derive shifts of humidity (~4%) and temperature (~−1 K) within the first 0.5 h, which may have contributed to the observed peak in resonance frequency shift (~−1.7 Hz) and mass concentration (~16 µg/m^3^, Figure 11a). Furthermore, we have to discuss the considerably increased deviations in the second half of the exposure-measurement period. Here, the battery that powers the fan was fortuitously drained, and thus, the forced airflow towards the cantilever was stopped after running for 3.5 h. Supposedly, this effect resulted in a smaller total volume of sample air, which was laden with particles fluctuating in size according to the distribution shown in Figure 9a. This can be expected to lead to an increased uncertainty of the adsorbed mass on the cantilever [37]. On the other hand, surface stress on the cantilever beam due to a fluid drag force was released, omitting its effect on resonance frequency [39]. A further source of error could be the loss of large line-shape agglomerates, which preferentially form at the edges of the cantilever. Because of their larger inertia (vs. single particles), they may be prone to detach during resonance oscillation of the cantilever. This would explain the sudden decrease in the mass concentration measured with the EtPCS after ~5.5 h.

**Figure 12:**
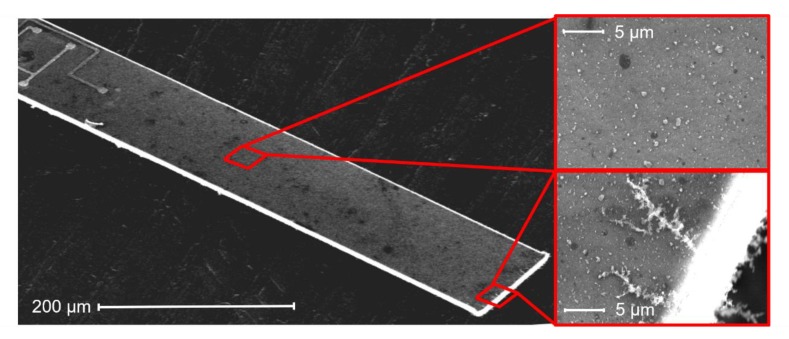
Surface morphology of the sampling area of the EtPCS after carbon-nanoparticle exposure. The deposited carbon nanoparticles are uniformly distributed across the area, while particle agglomerates are trapped on the cantilever edge.

Figure 12 shows a scanning electron microscope (SEM) image of an enlarged section of the cantilever surface, in which we find a distribution of deposited nanoparticles with different sizes and patterns. Furthermore, some big particle agglomerations were found mostly on the edge of the cantilever. A larger concentration of aerosolized particles in overall could increase the particle-agglomeration potential. With a greater concentration of particles in the aerosol, the propensity for particle-to-particle attraction and agglomeration is increased when compared to lower concentrations [40].

To estimate the limit of detection (LOD) of the EtPCS sensor, we operated the sensor under constant temperature (*T* = 22.3 ± 0.4 °C) and relative humidity (*rH* = 23 ± 1%) as depicted in Figure 11b and measured *σ* = 0.05 ± 0.009 Hz (baseline frequency noise averaged over the *t*_CT_ = 10 min). An LOD of 1.4 ± 0.3 µg/m^3^ was calculated using Equation (2) and *CF* = 96 µg × min/(m^3^ × Hz). By employing the MFLI instrument and our optimized PLL system, we have evidently suppressed the baseline frequency noise dramatically and improved the LOD, compared to previous work (~16 µg/m^3^) [27]. Nevertheless, working with a reduced high voltage for particle collection, i.e., from −500 V to −115 V, may have decreased the particle-collection efficiency corresponding to a much lower average frequency shift rate of 0.17 ± 0.02 Hz/min in the present study with respect to the previously measured 2.3 Hz/min at similar mass concentrations [27].

### 3.2. AFM-MCS Cantilever Performance

**Figure 13:**
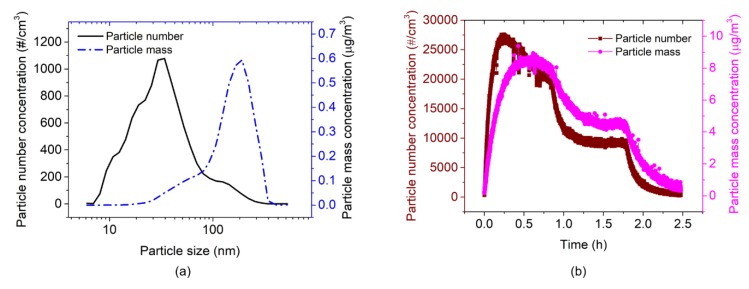
(**a**) Typical carbon-nanoparticle number and mass concentrations depending on the particle size and (**b**) total particle number/mass concentrations measured by FMPS.

The AFM-MCS was operated without extra airflow around the cantilever during particle sampling. Here, the movement of carbon nanoparticles towards the cantilever can only be expected to be courtesy of the ventilation fan inside the chamber. An FMPS measurement of nanoparticle exposure (for ~2.5 h) is shown in Figure 13. The typical size distributions of particle number and mass concentrations (shown in Figure 13a) reveal average particle sizes of ~34 nm and ~191 nm, respectively, which are similar to the measurement run with the EtPCS (cf. Figure 9a). Total number (~27,000 #/cm^3^ at the peak) and corresponding mass concentrations (Equation (3), ~8.6 µg/m^3^ at the peak) are delineated in Figure 13b. 

**Figure 14:**
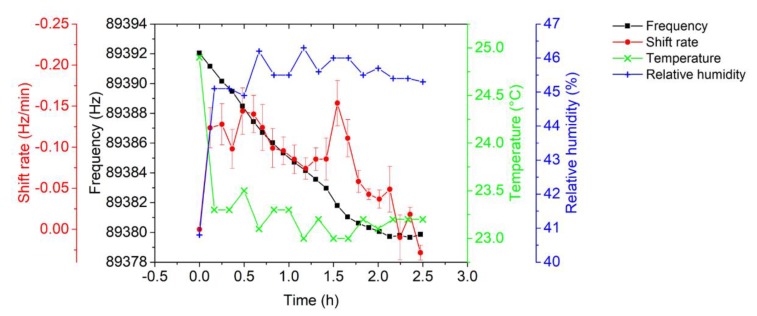
Decrement in resonance frequency (taken at a phase of 72°) and the corresponding frequency shift rate at *t*_CT_ ≈ 7 min and ambient conditions (*T* = 23.3 ± 0.23 °C; *rH* = 45.3 ± 0.63%).

By interpolating the amplitude and phase responses, the resonance frequency was determined at a phase of 72°, which corresponds to the maximum amplitude (~19 mV). The approximated resonance-frequency decrement due to particle collecting measured by the AFM-MCS and the corresponding frequency-shift rate over *t*_CT_ = 7.07 ± 0.39 min are plotted in Figure 14 with an average shift rate of 0.08 ± 0.04 Hz/min. Simultaneously, fluctuations of temperature and relative humidity are slightly on the same level as in the previous EtCPS measurements, i.e., ±0.2 °C and ±0.6%, respectively.

**Figure 15:**
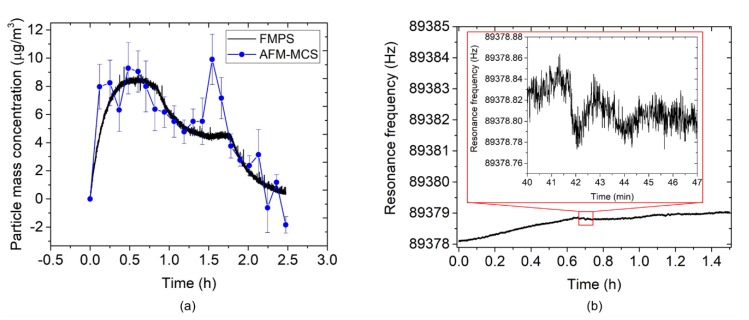
(**a**) Carbon-nanoparticle mass concentration measured by the AFM-MCS (full blue circle) in comparison with FMPS (full black line). (**b**) The frequency-stability measurement under a temperature of 23.01 ± 0.03 °C and relative humidity of 21.8 ± 0.4%. The inset shows an exemplary detailed enlargement of the frequency tracking within 7 min showing *σ* ≈ 17 mHz.

**Figure 16:**
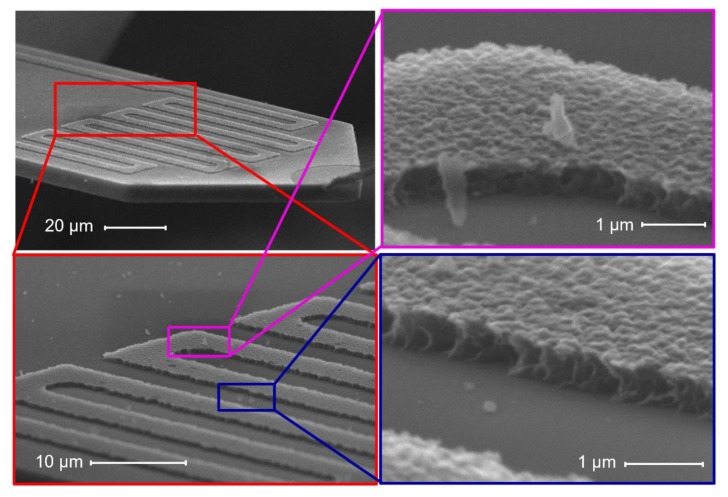
Surface morphology of the sampling area of the AFM-MCS cantilever after carbon-particle exposure. Particles deposited on the rough surface of the meander resistor are hardly detectable, while few particles were trapped around it.

The calculated mass concentration values by the AFM-MCS in comparison with the FMPS is depicted in Figure 15a. In this case, Equation (1) is used with *CF* = 64.5 µg × min/(m^3^ × Hz). The average uncertainty amounts to 1.3 ± 0.5 µg/m^3^, and the deviation from the FMPS measurement was 28.7 ± 9.4%. Here, the fluctuation of particle-size distribution sampled on the cantilever due to the small size of the collection area is most likely the main factor to this measurement error (e.g., for the two outliers at ~1.5 h). In addition, the absence of forced airflow towards the AFM-MCS cantilever will have affected the total amount of sampled particles. The SEM images depicted in Figure 16 show that particles of different sizes and shapes were sparingly spread around the meander resistor. However, it is difficult to find particles on the resistor owing to the high roughness on the surface of its covering layer (aluminum oxide). Furthermore, compared to the EtPCS sensor, large agglomerates were not found on the AFM-MCS cantilever, possibly due to the lower carbon particle concentration selected in this experiment.

Furthermore, a baseline frequency noise of the AFM-MCS of *σ* = 0.027 ± 0.004 Hz was measured by track its resonance within a *t*_CT_ ≈ 7 min (inset Figure 15b), which subsequently yields an LOD of 0.7 ± 0.1 µg/m^3^ using Equation (2) and *CF* = 64.5 µg × min/(m^3^ × Hz). If we compare the LOD of the EtPCS to that of the AFM-MCS, the latter has a considerably smaller frequency-noise floor that effectively results in a lower LOD value. Furthermore, although particle collection on AFM-MCS was performed without extra airflow, a very good average particle collection rate (0.08 ± 0.05 Hz/min) was achieved on a much smaller particle-collection area and at a lower particle concentration. It indicates that AFM-MCS has nearly the same resolution for piezoresistive cantilever sensor of even lower mass (~5 ng) [37]. Nevertheless, the cantilever size downscaling, as in the case of the AFM-MCS sensor, presents difficulties in handling and regeneration by cleaning from deposited particles. Moreover, it also leads to a smaller surface area for particle sampling, and hence, a much lower probability of sampling target particles on the sensor surface.

In Table 1, we show and compare various important, relevant parameters (i.e., particle sampling, air-flow rate, resonance frequency, sensitivity, particle sampling rate, collecting time, and LOD) in this work to other types of piezoresistive resonators, i.e., dual-plate thermal-piezoresistive resonator (DP-TPR) and thermal-piezoresistive oscillator (TPO). From this comparison, it is evident that EtPCS (*f*_0_ ~ 200 kHz) and AFM-MCS (*f*_0_ ~ 89 kHz) devices have lower sensitivities. Nevertheless, using such simple cantilever structures, we have demonstrated and achieved a fairly competitive resolution value (LOD) with only 7–10 min of particle collection time in the electrophoresis setup. Compared to the inertial impaction technique, the electrophoretic method could work in low particle velocity, and no large vacuum pump is required. However, to enhance the efficiency in the particle sampling rate of the EtPCS, the voltage difference between the copper ring and the EtPCS cantilever beam is necessary to be increased. While the smaller distance between the AFM-MCS cantilever beam and the metal electrode is a more crucial issue, its therefore necessary to improve the electrophoresis setup. In addition, the presence of consistent airflow (which acts as a particle deliverer) is also essential in nanoparticle mass detection.

## 4. Conclusions

Measurement of airborne-nanoparticle mass concentrations using two kinds of piezoresistive microcantilever sensors (electrothermal piezoresistive cantilever sensor (EtPCS) and tip-less atomic force microscopy micro-cantilever sensor (AFM-MCS)) has been presented. By operating in an in-plane mode, the EtPCS cantilever resonates with a higher *Q* factor (~2000) compared to the AFM-MCS cantilever (Q ~189), which operates in out-of-plane mode. Furthermore, in regard to the nanoparticle measurement, in principle, both sensors showed a good agreement with the FMPS reference measurement under defined concentrations of carbon nanoparticles. Particles were collected by electro-/dielectrophoresis on the cantilevers’ surfaces, as evidently observed by SEM inspection. Average deviations of 24.9 ± 0.3% and 28.7 ± 0.4% from the reference mass concentrations (fast mobility particle sizer—FMPS) were achieved for the EtPCS and the AFM-MCS, respectively. Moreover, average uncertainties of ~2.3% and ~1.3% were realized using the EtPCS and the AFM-MCS, respectively. The limits of detection (LOD) of ~1.4 µg/m^3^ were determined using the EtPCS and ~0.7 µg/m^3^ with the AFM-MCS cantilever. Measurement error with respect to FMPS of the AFM-MCS was considered to be mostly caused by fluctuations of the particles-size distribution in the air around the collection area, which has much smaller size than in case of the EtCPS. Furthermore, fluctuations of particle-size distribution may have caused the increased measurement error with the EtPCS when a forced flow of particle-laden air towards the cantilever was not active. Therefore, both airflow and particle-sampling high voltage (HV) are the most important factors for further improvement of miniaturized gravimetric nanoparticle sensors. 

## Figures and Tables

**Table 1:** Comparison of different piezoresistive gravimetric sensor approaches.

Reference	EtPCS Cantilever (This Work)	AFM-MCS Cantilever (This Work)	DP-TPR [41]	TPO [42]
Particle sampling	electrophoresis (−115 V), continuous	electrophoresis (−115 V), intermittent with frequency sweeping	chip-scale single inertial impactor	inertial impactor, intermittent with frequency tracking
Air-flow rate	680 mL/min	0	<0.1–0.25 mL/min	−
Resonance frequency	201.54 kHz	89.38 kHz	5.26 MHz	~950 kHz
Mass sensitivity	0.013 Hz/pg	0.14 Hz/pg	42 Hz/pg	1.946 Hz/pg
Particle collection efficiency	0.12%	− (no air-flow)	100% *	−
Collecting time	10 min	7 min	60 min	5 min
LOD	1.4 µg/m^3^	0.7 µg/m^3^	0.01–0.2 µg/m^3^ **	50 µg/m^3^

* assumed for calculating particle mass concentration. ** calculated assuming 100% particle collection efficiency (not verified by reference measurements).
